# Decontamination of bedding reduces the risk for contamination of personnel changing bedding: A simulation study

**DOI:** 10.1017/ash.2023.312

**Published:** 2023-09-29

**Authors:** Jennifer Cadnum, Andrew Osborne, Samir Memic, Curtis Donskey, Maria Torres-Teran

## Abstract

**Background:** The recent worldwide outbreak of Mpox virus infections has raised concern about the potential for nosocomial acquisition during handling of contaminated bedding or clothing. We conducted simulations to test the hypothesis that decontamination of bedding prior to handling could reduce the risk for contamination of personnel. **Methods:** We conducted a crossover trial to test the effectiveness of spraying contaminated bedding with a hydrogen peroxide disinfectant in reducing contamination of personnel during handling of the contaminated bedding. Bedding was contaminated on top and bottom surfaces with aerosolized bacteriophage MS2. Personnel (N = 10) wearing a cover gown and gloves removed the bedding from a patient bed and placed it into a hamper both with and without prior hydrogen peroxide spray decontamination. After handling the bedding, samples were collected to assess viral contamination of gloves, cover gown, neck or chest, and hands or wrists. **Results:** Contamination of the gloves and cover gown of personnel occurred frequently during handling of bedding and 20% of participants had contamination of their hands or wrists and neck after the simulation (Fig.). Decontamination of the bedding reduced contamination of the gloves and eliminated contamination of the cover gown, hands or wrists, or neck. **Conclusion:** Decontamination of bedding prior to handling could be an effective strategy to reduce the risk for nosocomial acquisition of Mpox by healthcare personnel.

**Figure:**
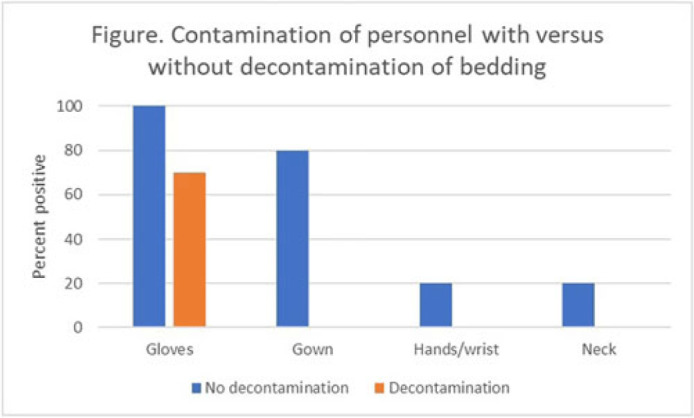

**Disclosures:** None

